# A New Natural Antioxidant Mixture Protects against Oxidative and DNA Damage in Endothelial Cell Exposed to Low-Dose Irradiation

**DOI:** 10.1155/2017/9085947

**Published:** 2017-08-09

**Authors:** T. Cervelli, D. Panetta, T. Navarra, S. Gadhiri, P. Salvadori, A. Galli, D. Caramella, G. Basta, E. Picano, S. Del Turco

**Affiliations:** ^1^Institute of Clinical Physiology, Council of National Research (CNR), Via Moruzzi, 1, 56124 Pisa, Italy; ^2^Radiologia Diagnostica e Interventistica, Università di Pisa, Pisa, Italy

## Abstract

Exposure to ionizing radiation during diagnostic procedures increases systemic oxidative stress and predisposes to higher risk of cancer and cardiovascular disease development. Many studies indicated that antioxidants protect against radiation-induced damage and have high efficacy and lack of toxicity in preventing radiation exposure damages. The purpose of this study was to investigate the *in vitro* protective effect of a new antioxidant mixture, named RiduROS, on oxidative stress generation and DNA double-strand breaks (DSBs) induced by low doses of X-rays in endothelial cells. Human umbilical vein endothelial cells (HUVEC) were treated with RiduROS mixture 24 h before a single exposure to X-rays at an absorbed dose of 0.25 Gy. The production of reactive oxygen species (ROS) was evaluated by fluorescent dye staining and nitric oxide (NO) by the Griess reaction, and DSBs were evaluated as number of *γ*-H2AX foci. We demonstrated that antioxidant mixture reduced oxidative stress induced by low dose of X-ray irradiation and that RiduROS pretreatment is more effective in protecting against radiation-induced oxidative stress than single antioxidants. Moreover, RiduROS mixture is able to reduce *γ*-H2AX foci formation after low-dose X-ray exposure. The texted mixture of antioxidants significantly reduced oxidative stress and *γ*-H2AX foci formation in endothelial cells exposed to low-dose irradiation. These results suggest that RiduROS could have a role as an effective radioprotectant against low-dose damaging effects.

## 1. Introduction

The employment of medical imaging diagnostic procedures delivering relatively low doses of ionizing radiation (IR) has never stop to grow since the last years [1, 2]. Despite many clinical advantages, exposure IR, even at low-doses, may cause damage at biological systems and predispose individuals at an increasing risk of developing cancer and cardiovascular diseases (CVD) [3–5]. Low doses of IR induce oxidative stress [6–8]. The excessive accumulation of ROS may provoke structural modifications to biological systems inducing cellular damage [9, 10]. DNA double-strand breaks (DSBs) are the most serious consequence of free radicals after radiation exposure [11] and, if inefficiently repaired, can lead to carcinogenesis and predispose to vascular aging processes [12, 13].

Endothelium seems to play an important role in the cardiovascular response to ionizing radiation. Alterations of endothelial function induced by low-dose irradiation increases the risk of CVD [14]. We have previously showed that low doses of X-ray irradiation can induce oxidative stress and DSBs in human endothelial cells predisposing to accelerate vascular inflammation, from which the atherosclerotic process can arise [15].

Recently, there has been a growing interest in research into the protective role of antioxidant agents against radiation-induced oxidative stress [16]. ROS-mediated harmful effects may be promptly quenched not only by endogenous defense mechanisms but also by taking supplements of antioxidants that are able to quench oxidative stress caused by ionizing radiation, therefore reducing the potential risks for human health [16]. *In vitro* and animal studies [17–20] have demonstrated the efficacy of multiple antioxidants in the protection of tissues from damage induced by ionizing radiation.

Since increased ROS generation occurs in different cellular compartments inducing cell damage at multiple levels, the use of combined antioxidants would represent a more effective strategy in protecting against radiation damage than single antioxidant [21].

Focusing on this, a new mixture of antioxidants, RiduROS, has been developed selecting nutrients or molecules such as resveratrol, Extramel®, seleno-L-methionine, *Curcuma longa*, reduced L-glutathione, and vitamin C that are well-known for their antioxidant capacity. Curcumin, a phenolic compound from the rhizomes of *Curcuma longa*, is a powerful scavenger of superoxide anion [22] and shows protective effects against radiation-induced damage [23], reducing oxidative stress, inflammatory response [24], and DNA damage [25]. Resveratrol, a natural polyphenol compound [26] with reported antioxidant and anticarcinogenic effects, has received particular interest as a radioprotector with the potential for widespread application [27, 28]. L-selenium methionine is an essential component of several antioxidant enzymes that does not act as a free radical scavenger, but acts indirectly providing protection against oxidative stress. Reduced L-glutathione [29] and vitamin C [30] have been observed to mitigate the DNA damage induced by ionizing radiation by scavenging reactive oxygen species. Extramel is a melon juice concentrate rich in superoxide dismutase (SOD), with potential beneficial effects on the development of atherosclerosis and liver steatosis, characterized by an increased oxidative stress and chronic inflammation [31]. SOD is an endogenous antioxidant enzyme that can confer radioresistance [32] or radiosensitivity [33], whether over- or downexpressed, respectively, and it has been successfully used as a treatment in chronic damage induced by radiotherapy in humans [34, 35].

The objectives of this study were to evaluate the effect of RiduROS mixture on oxidative stress induced by low-dose X-ray in human endothelial cells and to show its effect on DSBs, one form of DNA damage induced by oxidative stress.

## 2. Material and Methods

### 2.1. RiduROS Composition

RiduROS is composed by a mixture of antioxidants in different percentages (weigh percent, %) as follows: resveratrol (45%), Extramel (2.15%), seleno-L-methionine (2%), *Curcuma longa* (42%), reduced L-glutathione (6%), and vitamin C (2.4%) (supplied by BRG Farmaceutica, Grosseto, Italy).

RiduROS was soluble in dimethyl sulfoxide (DMSO), an organic solvent, at a concentration of 20 mg/mL. Further dilutions of the mixture were performed in culture medium to reach the specified final concentrations for the experiments. The same batch of RiduROS was tested in all experiments, as provided by BRG. Contribution of each compound to antioxidant activity was tested at one concentration only—the equivalent of its concentration in 1 *μ*g/mL RiduROS.

### 2.2. X-Ray Irradiation Setup and Calibration

The experimental setup for X-ray irradiation consists of a system aligned on a vertical axis, with variable source-to-object distance in the range of 90–375 mm. Polystyrene Petri dishes or microwell plates (Corning/Costar Inc., Cambridge, USA) are placed above a 1 cm-thick block of polymethyl methacrylate (PMMA) [15]. For operator safety, the whole system is enclosed in a shielded cabinet (<1 *μ*Sv/h at each accessible point during operation). The X-ray source (Apogee, Oxford Instruments, USA) is a packaged X-ray tube with fixed tungsten anode and 125 *μ*m-thick beryllium window, operating in continuous mode. An additional 1 mm-thick aluminium filter was placed in front of the X-ray exit window. The maximum accelerating voltage and filament current are 50 kV and 1 mA, respectively (50 W max. continuous power).

The X-ray tube is operated by a general purpose personal computer through a dedicated controller with RS232 interface (ADIO232, RFS Systems, Straubenhardt, Germany). The average irradiation time for a total dose of 0.25 Gy was 164 s, corresponding to a dose rate of 91 mGy/min.

For all the experiments, the irradiation parameters were 50 kV, 0.7 mA, source-to-object distance = 211 mm. Thermoluminescent dosimeters (TLD-100, Harshaw) at the same position of the specimen were used before experiments to calibrate the system in term of absorbed dose.

### 2.3. Endothelial Cell Culture and Experimental Design

Human umbilical vein endothelial cells (HUVEC) were harvested and isolated by enzymatic digestion in the presence of type II collagenase (0.1%) as described before [36]. Isolated cells were maintained in Medium 199 (Life Sciences, Grand Island, NY, USA), containing fetal bovine serum (10%), antibiotics, and growth factors (heparin, 50 U/mL, and endothelial cell growth factor, 10 mg/mL) (all from Sigma-Aldrich, St. Louis, MO, USA). Human cells were obtained from discarded umbilical cords and treated anonymously; as such, approval from the University Ethics Review Board was not necessary. HUVEC were used at passage 2 after primary culture. Cell monolayers were pretreated with RiduROS (0.1, 1, and 10 *μ*g/mL) or a single compound for 24 h before exposure to a single final dose of 0.25 Gy X-ray radiations with a dose corresponding to maximum oxidative stress generation compared with the control, as previously demonstrated [15]. Incubation with only DMSO (0.0075%, the final percent to reach the concentration of RiduROS 1 *μ*g/mL) was run in parallel, and no inhibition of oxidative stress was observed with DMSO after irradiation. Control cells were treated exactly as irradiated samples, except for irradiation and/or drug treatments.

### 2.4. Cell Viability Assay

HUVEC, seeded in 96-well microplates, were treated with RiduROS (0.1, 1, and 10 *μ*g/mL) and the effects on cell viability was monitored at 24 and 48 h after treatment. The viability of HUVEC was assayed by colorimetric assay using WST-1 (Biovision, San Francisco, USA) which was based on cleavage in viable cells of the water-soluble tetrazolium salt WST-1 [2-(4-iodophenyl)-3-(4-nitrophenyl)-5-(2,4-disul-fonyl)-2H-tetrazolium, monosodium salt] to a formazan dye by mitochondrial dehydrogenase. Briefly, cells cultured in 96-well plates were treated with different concentrations of RiduROS or a single antioxidant component for 24–48 h. Then, 10 *μ*L WST-1 reagent was added to each well and HUVEC were incubated for 4 h at 37°C. The formazan dye produced was quantified by measuring the absorbance of the dye solution at 450 nm with a microplate reader.

### 2.5. Detection of Intracellular ROS Generation

Generation of ROS in HUVEC was measured with 25 *μ*mol/L of the fluorescent dye 6-carboxy-2′,7′-dichlorodihydrofluorescein diacetate bis(acetoxymethyl)-ester (C-DCDHF-DA) (Molecular Probes Inc., Eugene, OR, USA), which is a cell-permeable nonfluorescent probe that, after uptake, is cleaved by intracellular esterases to carboxy dichlorofluorescein. In this status, C-DCDHF-DA is trapped within the cells and oxidized by ROS to highly fluorescent product. The fluorescence intensity is proportional to the level of intracellular oxidative stress [37]. Specifically, HUVEC, treated as described above, were washed with phenol red-free Hanks'-buffered saline (Sigma), treated with C-DCDHF-DA for 30 min at 37°C, and then irradiated. Forty-five minutes after irradiation, ROS generation was evaluated; cells were washed and scraped off into 1 mL of distilled water, sonicated, and centrifuged. The fluorescence of supernatants was measured with a spectrofluorometer at 485 nm excitation and 525 nm emission.

### 2.6. Determination of Nitrite/Nitrate Production

HUVEC were treated as described above, supernatants were collected, and total levels of nitrate plus nitrite, the final products of nitric oxide, were measured with the Griess assay kit (Cayman Chemical Co., Ann Arbor, MI, USA). Concentration of NO_2_^−^/NO_3_^−^ was corrected for the cell number and expressed as *μ*mol/L/10^5^ cells [38].

### 2.7. Immunofluorescence Microscopy and Semiquantification of *γ*H2AX Foci

The number of phosphorylated *γ*-H2AX foci in cell nuclei is an efficient marker for scoring radiation-induced DSBs [36, 39]. HUVEC were seeded on glass cover slips at 80% of confluence the day before treatment in order to have a monolayer the day of irradiation. The cells were fixed with 2% paraformaldehyde 2 h after irradiation and treated as previously described [36]. The primary antibody against *γ*-H2AX (anti-phospho-Histone H2AX (Ser139), clone JBW301, Millipore) was diluted at 1 : 200 in PBS containing BSA/glycine. The secondary antibody tagged to a fluorescent group (Alexa Fluor 594 goat *α*-mouse IgG, Thermo Fisher Scientific) was applied diluted at 1 : 500 in PBS with BSA/glycine. Cover slips were put on object glasses covered with DAPI/Vectashield and sealed.

Analysis of foci formation was performed using a ZEISS Axioskop40 fluorescence microscope equipped with a ×100 magnification objective. In each sample, the number of foci was counted in 100 cells. The number of foci/cell was determined by the ratio between total number of foci and total enumerated cells.

### 2.8. Statistical Analysis

Two-group comparisons were performed by unpaired Student's *t*-test. Multiple comparisons were performed by one-way analysis of variance (ANOVA) followed by a multiple comparison test (Bonferroni test). The percent of inhibition was normalized for control cells. Values of *P* < 0.05 were considered statistically significant.

## 3. Results

**Figure 1:**
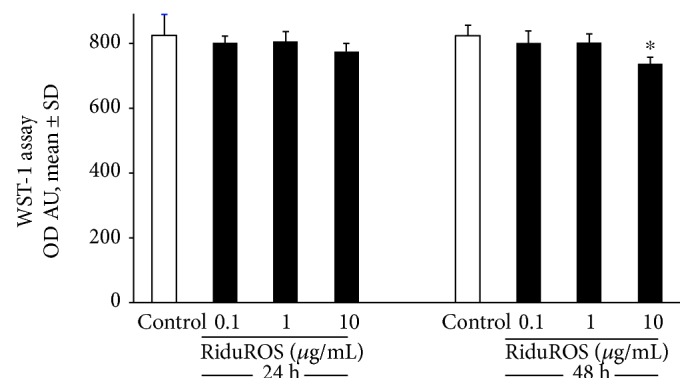
Effect of RiduROS on endothelial cell viability. Cell viability was assessed by WST-1 24–48 h after treatment with RiduROS (0.1, 1, and 10 *μ*g/mL). Data are expressed as mean ± SD of optical density (OD) arbitrary units at 450 nm and are representatives of three independent experiments. ^∗^*P* < 0.05 versus control cells.

**Figure 2:**
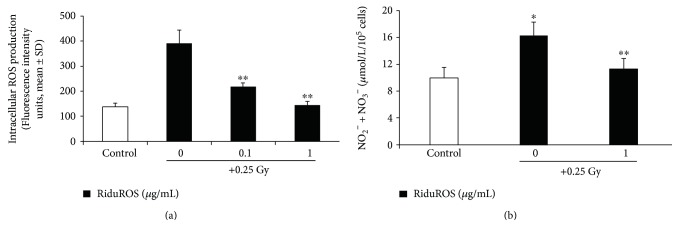
RiduROS inhibited ROS generation and restore NO levels after low-dose irradiation. (a) HUVEC were pretreated with RiduROS (0.1–1 *μ*g/mL), then exposed to 0.25 Gy of radiation. After 45 min of incubation, monolayers were harvested and lysed for quantitative determination of ROS. All values are expressed as mean ± SD of arbitrary fluorescence units of three independent experiments. ^∗∗^*P* < 0.001 versus 0.25 Gy alone. (b) Supernatants were collected 24 h after irradiation and total levels of nitrate/nitrite were measured. Data are mean ± SD and representatives of three independent experiments and expressed as *μ*mol/L/10^5^ cells. ^∗^*P* < 0.05 versus control cells; ^∗∗^*P* < 0.05 versus 0.25 Gy alone.

The main objective of this study was to assess RiduROS ability in protecting endothelial cells from oxidative and DNA damage induced by exposure to low dose of X-rays. It was observed that the treatment with RiduROS mixture (0.1, 1, and 10 *μ*g/mL) did not affect cell viability up to 1 *μ*g/mL at 24–48 hours after treatment, as shown in Figure 1. At 10 *μ*g/mL, we observed a weak reduction in cell viability after 24 h of treatment (10%) that increases significantly after 48 h (16%). This decrease, probably, is due to coadministration and high concentrations of antioxidants that augment the clearance of physiologically levels of free radicals, which are essential to regulate intracellular signaling processes and to guarantee the structural integrity of cellular components. The application of antioxidant mixture 24 hours before irradiation proved to be the most effective incubation time in reducing oxidative stress compared to 1 h (97% versus 18%, data not shown), compatible with appropriated delivery and distribution of antioxidants inside cells. So, all experiments were performed pretreating HUVEC with RiduROS for 24 h. Oxidative stress was evaluated 45 minutes after irradiation with 0.25 Gy X-rays, corresponding to the time of maximum increase in the ROS generation [15]. Results showed that the pretreatment with the antioxidant mixture blunted ROS generation in a concentration-dependent manner by 65% ± 5.6% and 98% ± 2%, at 0.1 and 1 *μ*g/mL, respectively, compared with cells irradiated without pretreatment (Figure 2). The contribution of each substance to antioxidant activity, at concentrations equivalent to that in the RiduROS 1 *μ*g/mL, was also tested. As evident in Table 1, the resveratrol and *Curcuma longa* were highly protective against oxidative stress induced by low-dose irradiation with an inhibition percent of 57% and 79%, respectively. Extramel and L-seleno-L-methionine inhibit ROS generation by 48% and 41%, respectively. Conversely, vitamin C and reduced glutathione showed a lower antioxidant activity, decreasing oxidative stress by 29% and 39%, respectively **(**Table 1).

Once demonstrated that the RiduROS mixture suppresses oxidative stress, highlighting the advantage of using multiple antioxidants, we have also evaluated its effect both on NO levels and on number of *γ*-H2AX-foci induced by 0.25 Gy X-rays.

Low dose of X-rays reduce NO levels by 36%, measured as concentration of nitrite and nitrate, indicating an impairment of endothelial function, while RiduROS tends to restore NO within its physiological range (Figure 2(b)).

**Figure 3:**
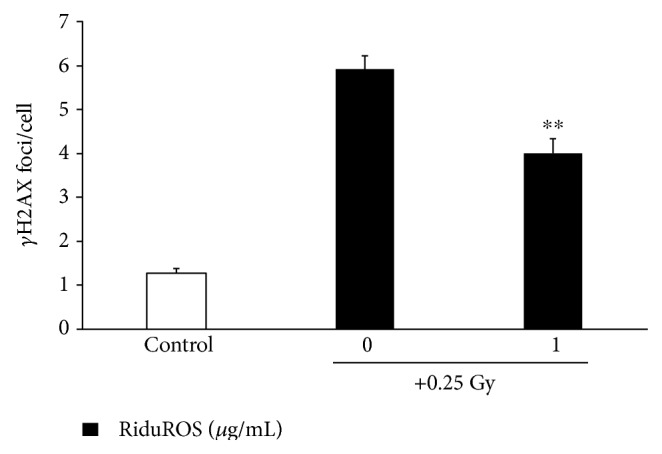
RiduROS reduced the number of *γ*-H2AX foci/cell after low-dose irradiation. HUVEC were pretreated with RiduROS (1 *μ*g/mL), then exposed to 0.25 Gy of radiation and fixed after 2 h. All values are expressed as mean ± SD of foci/cell of three independent experiments. ^∗∗^*P* < 0.05 versus 0.25 Gy alone.

As shown in Figure 3, low-dose irradiation increased DSB-induced *γ*-H2AX foci compared with control cells and 24 h of RiduROS pretreatment reduces of 41% the *γ*-H2AX foci number, therefore providing a beneficial effect on DNA damage.

## 4. Discussion

The increasing exposure to radiation associated to diagnostic procedures requires insight into human health risks, especially in terms of carcinogenic and cardiovascular risks [40, 41]. Low doses of ionizing radiation used in diagnostic imaging procedures can produce acute and long-term side effects by free radicals that affect DNA and biological molecules ultimately resulting in molecular and biochemical alterations [42]. DSBs are considered the most significant DNA lesions induced by ionizing radiation [43]. We have previously demonstrated that low doses of X-rays are capable of inducing free radical generation and DSBs in human endothelial cells [15].

The present study was designed to investigate the protective effects of a new mixture of antioxidants on oxidative and DNA damage-induced by X-ray irradiation at low dose.

At present, the development of novel and effective agents to combat radiation damages is of considerable interest particularly in radio diagnostics. To quench the effect of free radicals, antioxidant treatment may be useful in reducing radiation-related adverse effects. Ionizing radiation produces many types of free radicals that interact with various cellular targets. As antioxidants have multiple distribution/localization into the cell and different binding activities for free radicals, the use of a mixture of multiple antioxidants may result more effective in decreasing the oxidative stress than individual agents themselves.

Both *in vitro* and *in vivo* studies on animal models have demonstrated the beneficial effect of antioxidants on damage induced by ionizing radiation. In particular, the combined use of more than one antioxidant, such as vitamin E and selenium [17] or vitamins C and E [44], turned out to be more effective in reducing radiation-induced mutations and chromosomal damage than individual agents. These results support animal data, showing that a mixture of multiple antioxidants (i.e., sodium ascorbate, N-acetyl cysteine, *α*-lipoic acid, and coenzyme Q10 vitamins) protected mice against oxidative stress [45]. Studies in multiple antioxidant research indicate a reduction of the chromosomal and oxidative damage in subjects undergoing radiotherapy [16, 46] and in children chronically exposed to low doses of radiation in the Chernobyl area [47]. N-acetylcysteine, a naturally occurring compound found in several vegetables and one of the least toxic thiol reducing agents, may be an effective mean of preventing DNA damage induced by ionizing radiation exposure as during cardiac catheterization procedures [48]. A formulation of antioxidants and glutathione-elevating enzymes reduces the amount of X-ray–induced *γ*-H2AX at a typical dose for a computed tomography scan and other radiographic or scintigraphic studies [49].

However, at present, the efficacy of combined antioxidant strategy to reduce the side effects of low doses of X-rays, as administered during diagnostic procedures, has not yet been tested in humans.

Our results have demonstrated that all antioxidants of RiduROS are able to reduce oxidative stress induced by low-dose irradiation and complete or nearly complete reduction was achieved with RiduROS mixture applied before the radiation exposure.

Moreover, ROS reduction was accompanied both by increase of NO concentration, by restoration of endothelial function, and by reduction of *γ*-H2AX foci supporting the efficacy of RiduROS treatment in protecting DNA from DSBs.

Since individuals are exposed to very low doses of ionizing radiation for early diagnosis of diseases, the development of useful and nontoxic agents to combat radiation damage and protect biological systems is of paramount importance. The results of our study suggest that the RiduROS could have a role as an effective radioprotector against low-dose damaging effects and considered in future clinical trials to evaluate the relevance for patients.

## Figures and Tables

**Table 1:** Effect of single antioxidant and RiduROS on ROS generation detected by dichlorofluorescein assay.

*Antioxidant component of RiduROS*	% of inhibition of oxidative stress
Resveratrol (1 *μ*M)	57% ± 8
Extramel melon pulp (0.00025 UI)	48% ± 5
Seleno-L-methionine (0.5 nM)	51% ± 6
*Curcuma longa* (225 nM)	79% ± 3
Reduced L-glutathione (0.25 *μ*M)	29% ± 5
Vitamin C (200 *μ*mol/L)	39% ± 6
RiduROS (1 *μ*g/mL)	98% ± 2
